# Transmission of tetracycline resistance genes and microbiomes from manure-borne black soldier fly larvae frass to rhizosphere soil and pakchoi endophytes

**DOI:** 10.3389/fmicb.2022.1014910

**Published:** 2022-10-31

**Authors:** Jingyuan Chen, Yingfeng Cai, Weikang Deng, Sicheng Xing, Xindi Liao

**Affiliations:** ^1^College of Animal Science, South China Agricultural University, Guangzhou, Guangdong, China; ^2^Guangdong Provincial Key Lab of Agro-Animal Genomics and Molecular Breeding, and Key Laboratory of Chicken Genetics, Breeding and Reproduction, Ministry Agriculture, Guangzhou, Guangdong, China; ^3^National-Local Joint Engineering Research Center for Livestock Breeding, Guangzhou, Guangdong, China

**Keywords:** black soldier fly, tetracycline, antibiotic resistance genes, rhizosphere, Pakchoi

## Abstract

Manure treatment with black soldier fly larvae (BSFL) and BSFL frass application in crop land is a sustainable strategy; however, whether residual antibiotic resistance genes (ARGs) and their transmission risk are related to the manure BSFL treatment process is still unknown. In this paper, the effect of BSFL addition density on residual tetracycline resistance genes (TRGs) and transmission from frass to pakchoi was determined. The results showed that BSFL frass can provide sufficient nutrients for growth, improve the economic value of pakchoi, and reduce the risk of transmission of TRGs in chicken manure regardless of BSFL density. The potential hosts of the TRGs we detected were found in BSFL frass (*Oblitimonas* and *Tissierella*), rhizosphere soil (*Mortierella* and *Fermentimonas*), and pakchoi endophytes (*Roseomonas*). The present study concluded that BSFL frass produced by adding 100 BSFL per 100 g of chicken manure has the advantages of high value and low risk. These findings will provide important strategic guidance for animal manure disposal and theoretical support for preventing the transmission of TRGs in BSFL applications.

## Introduction

In recent years, with continuous reforms and innovations related to waste recycling technology, the immeasurable value of black soldier fly (*Hermetia illucens*) larvae (BSFL) as a tool for manure disposal has gradually become apparent (Gold et al., 2018). Using insects to recycle manure is a sustainable solution (Miranda et al., 2021), and many studies have shown that BSFL can be used as protein feed, biodiesel, and antimicrobial peptides, among other products (Marco et al., 2015; Schiavone et al., 2017; Cutrignelli et al., 2018; Ruhnke et al., 2018). Compared with traditional aerobic composting and anaerobic digestion technologies with long processing times, high odor emissions, high environmental control requirements, and high input costs (Abouelenien et al., 2010; Lalander et al., 2015; Liu et al., 2021), the use of BSFL to treat manure has the advantages of a short processing time, more manure volume reduction, less odorous gas release, and low cost (Wang et al., 2013; Zhou et al., 2013; Lim et al., 2016; Beskin et al., 2018). As a sustainable resource recycling technology, BSFL composting may become a high-quality treatment method for manure that is difficult to fully dispose of in large areas of China. Many factors affect the efficiency of BSFL-mediated waste conversion, including rearing scales (Niu et al., 2022), feeding methods (Miranda et al., 2021), digested manure type (Wang et al., 2021), substrate humidity, and auxiliary materials (Bortolini et al., 2020). Although the factors affecting waste disposal by BSFL are complex, BSFL use is still a sustainable solution for recycling manure.

It is worth noting that, in manure treatment processes, antibiotic resistance gene (ARG) elimination efficiency has always been considered due to the biorisk of ARGs in the environment and organisms (Zhang et al., 2019; Gurmessa et al., 2021; Liu et al., 2021). There is substantial evidence that the dissemination of ARGs has become an urgent concern, especially, the use of antibiotics has made animals manure become a reservoir of antibiotic resistance genes (ARGs) in the farming industry(Fang et al., 2018; Karkman et al., 2018; Wang et al., 2018). Recent studies have shown that human pathogenic bacteria like *klebsiella* sp. can be used as hosts for ARGs(Niu et al., 2022; Wu et al., 2022), which undoubtedly strengthens the difficulty of treatment and involves global human public health and safety. In the process of BSFL digesting animal manure, the indigenous microorganisms in the BSFL gut inhibit the invasion of exogenous microorganisms and eliminate ARGs (Cai et al., 2018a; Wu et al., 2021); simultaneously, the flow of microorganisms between the BSFL gut and manure and the release of antimicrobial peptides by the BSFL gut reduces the abundance of pathogenic microorganisms and thus inhibits the horizontal spread of ARGs (Cui et al., 2017). However, BSFL cannot eliminate all ARGs (Liu et al., 2020), which directly leads to the risk of ARGs remaining in BSFL frass entering the soil and threatening human public health through the food chain (Zhang et al., 2019). Cai et al. (2018a) used approximately 2000 8-day-old BSFL cultured in 2000 g of fresh chicken manure for 12 days, effectively reducing ARGs by 95%. Niu et al. (2022) used four different BSFL addition densities to treat chicken manure and found that the addition density of 100 BSFL cultured in 100 g chicken manure was the best strategy for reducing the risk of ARGs. The studies above all show that the added density of BSFL will directly affect the conversion of manure, especially the reduction of manure-borne ARGs. However, they only focus on the quantification of ARGs in BSFL frass and do not consider the risk of ARG transmission from the perspective of the food chain. Although the application of organic fertilizers can cause ARG pollution in soil and plant tissues (Peng et al., 2015; Carpentieri et al., 2019; Zhang et al., 2019), the ARGs contained in the BSFL frass are unpredictable, whether ARGs in BSFL frass can pass through the food chain into soil and crops is unknown and urgently needs to be addressed.

It is well known that the ARGs are contaminants that originate in microorganisms (Yang et al., 2020), including bacteria and fungi. From the perspective of the food chain, during the application of BSFL frass as a soil nutrient, recent studies have only analyzed the bacterial community structure (Cai et al., 2018a, 2018b; Kawasaki et al., 2020; Zhang et al., 2020), ignoring the fungal community structure. Fungi are also one of the host microorganisms for ARGs (Nazir et al., 2017; Zhu et al., 2021), and even indirect factors of the fungal communities can alter ARG profiles by affecting bacterial communities (Zhu et al., 2021); thus, we should consider fungi when considering the risk of ARG transmission in BSFL frass. With the change in BSFL addition density, the physicochemical factors of BSFL frass also change (Miranda et al., 2021; Parodi et al., 2021); exploring the relationship between physicochemical factors, ARGs, the bacterial community, and the fungal community is the primary means to studying the risk assessment of ARG transmission.

In this study, we used pakchoi as the vegetable research object and established a glasshouse pot experiment to explore the transfer of tetracycline resistance genes (TRGs) in manure-borne BSFL frass to vegetables. We aimed to (i) explore the nutritional and economic value of pakchoi and the residues of ARGs after planting pakchoi with BSFL frass as fertilizer under different BSFL addition densities; (ii) explore potential bacterial and fungal TRG hosts; and (iii) select the optimal proportion of BSFL to add to sustainable economic strategies and provide reference data for human public health concerning BSFL frass application.

## Materials and methods

### Acquisition of manure, BSFL, soil, and pakchoi

Fresh manure from 150-day-old laying hens was collected from a local laying hen farm (Yunfu City, Guangdong Province). The collected manure was homogenized and stored at 20°C before thawing at room temperature for 24 h before use. The methods for BSFL acquisition and culture were based on the report by Niu et al. (2022). The soil samples used in the glasshouse experiment were purchased from a garden (Huainong Agriculture Co., Ltd., Huaian, China) and passed through a 2 mm sieve before use. The arable field has no history of organic fertilizer application. Pakchoi (*Brassica campestris L. ssp. Chinensis Makino*) seeds were purchased from a crop seed service center (Yifenghe Seed Service Department, Mianyang, China), and the planting cycle was 30 days.

### Pot experiment and processing

Three-day-old BSFL were collected and mixed with 50 kg of fresh laying hen manure in a plastic pool (150 cm × 200 cm × 60 cm) at different addition ratios (in the number of strips). Four treatments were established with four replicates: untreated manure without BSFL (CM), 50 BSFL cultured in 100 g of fresh manure (L), 100 BSFL cultured in 100 g of fresh manure (M), and 1,000 BSFL cultured in 100 g of fresh manure (H). The process ended when 50% of the BSFL entered the prepupa period. The postrearing BSFL frass was gathered and stored at-80°C for subsequent examination and the next phase.

**Figure 1:**
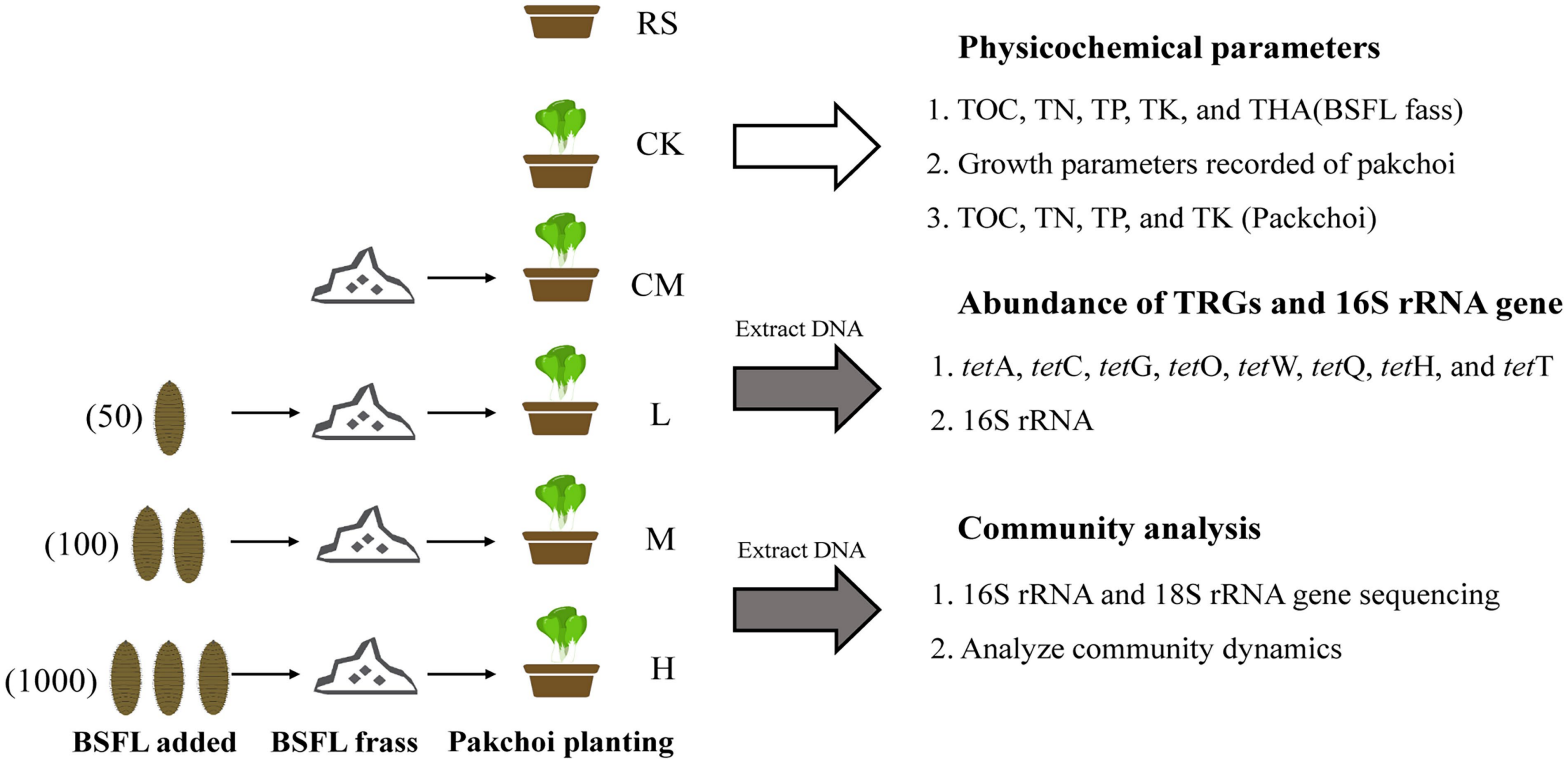
Experimental flow chart. “RS” means initial pure soil, “CK” means untreated soil, “CM” means untreated manure without BSFL, “L” means 50 BSFL cultured in 100 g of fresh manure, “M” means 100 BSFL cultured in 100 g of fresh manure, “H” means 1,000 BSFL cultured in 100 g of fresh manure.

The postrearing BSFL frass was thoroughly mixed with 2 l of soil in a plastic pot (33 cm × 12 cm × 13 cm) to grow pakchoi with four replicates while setting up a control group (CK). Pakchoi seeds were grown in sandy loam for germination without any fertilizer for 4 days, and then three pakchoi seedlings were transplanted into each pot. The pots were placed in a glasshouse located in South China Agriculture University, Guangzhou, Guangdong Province (23°9′39″N, 113°21′26″E). This area is located in the subtropical monsoon climate zone, with a monthly average temperature of 29.1°C, a monthly average humidity of 62.5%, a monthly rainfall of 189.9 mm, and an average monthly sunshine duration of 160.9 h. Plants were watered twice every day (7.30 a.m., 5.30 p.m.) with sterilized water to maintain 20% of the water-holding capacity and harvested at day 34 when the pakchoi reached maturity. The pakchoi samples were harvested separately by cutting at the soil surface using ethanol-sterilized scissors. The rhizosphere soil and the initial pure soil (RS) were also collected, and then all samples were stored at-80°C for subsequent examination. An experimental flow chart is shown in Figure 1.

### BSFL frass and pakchoi characteristics and DNA extraction

The following pakchoi growth parameters were recorded: height, leaf number, leaf length and width, weight, and moisture content. A portion of the pakchoi samples were dried at 105°C for 1 h after being rinsed with sterile water and then passed through a 40-mesh sieve for measurement.

The following characteristics of the pakchoi and BSFL frass samples were measured: total organic carbon (TOC), total nitrogen (TN), total phosphorus (TP), and total potassium (TK); simultaneously, BSFL frass samples were subjected to an additional assay to determine the total humic acid (THA), and pakchoi samples were additionally tested for growth indicators, including the leaf number, leaf length and width, height, fresh weight, and dry weight. TOC was determined by an elemental analyzer (ZA3300, Hitachi Limited, Japan). TN, TP, and TK were determined by the method described by Zhang et al. (2009), and total THA was measured following Li et al. (2014).

The DNA from BSFL frass (*n* = 16), rhizosphere soil (*n* = 24) and the endosphere of pakchoi (*n* = 20) was extracted. Microbial DNA in BSFL frass samples was extracted using a DNeasy PowerSoil Kit (Qiagen, Hilden, Germany) according to the manufacturer’s instructions. Microbial DNA in the rhizosphere soil and the endosphere of pakchoi were extracted using a FastDNA SPIN Kit (MP Biomedicals, United States) according to the manufacturer’s instructions. The pakchoi samples were treated as follows before DNA extraction: rinsing with sterile water within 24 h, soaking in 75% alcohol for 3 min, soaking in 10% sodium hypochlorite for 5 min to eliminate surface microorganisms, soaking in sterile water for 3 min in triplicate, cutting into small pieces with ethanol-sterilized scissors, mixing with liquid nitrogen in a sterilized mortar, and grinding thoroughly to extract DNA. All pure DNA was stored at-80°C for subsequent q-PCR, 16S rRNA, and 18S rRNA sequencing analyses.

### TRGs and bioinformatics analysis

The abundances of target genes were detected using qPCR (CFX96, BIO-RAD, United States). The genes included 8 TRGs (*tet*A, *tet*C, *tet*G, *tet*O, *tet*W, *tet*Q, *tet*H, and *tet*T) and 16S rRNA genes, and the primer sequences are shown in Supplementary Table S1. The absolute abundance of TRGs was calculated as described by Niu et al. (2022).

DNA samples were also used for bacterial 16S rRNA gene (V3-V4 region) and eukaryotic 18S rRNA gene (V4 region) sequencing analysis using the specific primers 338F/806R and 528F/706R with barcodes, respectively. Sequencing libraries were generated using Illumina TruSeq DNA PCR-Free Library Preparation Kit (Illumina, United States) following manufacturer’s recommendations and index codes were added. The library quality was assessed on the Qubit 2.0 Fluorometer (Thermo Scientific) and Agilent Bioanalyzer 2,100 system. At last, the library was sequenced on an Illumina NovaSeq platform and 250 bp paired-end reads were generated. Bioinformatic processing was performed using a combination of FLASH (V1.2.7)^1^ (Magoc and Salzberg, 2011), QIIME (V1.9.1)^2^ (Caporaso et al., 2010), and the UCHIME algorithm^3^ (Edgar et al., 2011). All samples were successfully sequenced and used for statistical analyses.

### Statistical analysis

All data, including the physicochemical data and qPCR results, were analyzed in Microsoft Excel 2019 (Microsoft, USA) with statistical significance determined with SPSS 22.0 (IBM Corp, USA), and the results were visualized with GraphPad Prism 8.0 software. Gephi (version 0.9.2) was employed for network analysis to evaluate the relationship between TRGs and bacterial genera based on Pearson’s correlation coefficients (the significance of the pakchoi sample was less than 0.05 and that of the rest of the samples was less than 0.01). The heatmap in the present study was generated using R software (version 4.1.3). A redundancy analysis (RDA) was conducted with CANOCO (version 5). The best larval density group analysis was obtained using the combination of a multifactorial pie scale diagram with Origin (version 9.8.0.200) software.

## Results

### Physicochemical parameters of BSFL frass and pakchoi samples

**Figure 2:**
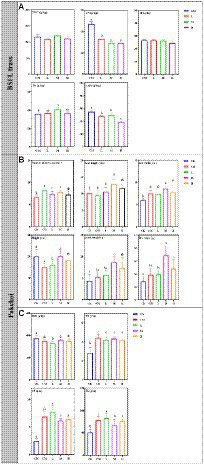
Physicochemical parameters of BSFL frass and pakchoi samples. **(A)** The contents of TOC, TN, TP, TK, and THA in BSFL frass among different treatments; **(B)** the leaf number, leaf length and width, height, fresh weight and dry weight of pakchoi among different treatments; **(C)** the contents of TOC, TN, TP, and TK in pakchoi among different treatments. Significant differences between the means were determined by Tukey’s test. Differences were considered significant when *p* < 0.05, and different lowercase letters indicate significant differences. “CK” means untreated soil, “CM” means untreated manure without BSFL, “L” means 50 BSFL cultured in 100 g of fresh manure, “M” means 100 BSFL cultured in 100 g of fresh manure, “H” means 1,000 BSFL cultured in 100 g of fresh manure.

The physicochemical parameters of BSFL frass and pakchoi were tested to reflect the value of BSFL frass as a substitute for chicken manure. The physicochemical parameters of BSFL frass are shown in Figure 2A. The content of TOC in chicken manure (216.91–240.15 g/kg) and TP (24.35–26.50 g/kg) was not altered by BSFL treatment, while the content of THA and TN gradually decreased with BSFL treatment and showed lower levels in H. The content of TK in BSFL frass in M was significantly higher than that in chicken manure in CM.

The growth parameters of pakchoi are shown in Figure 2B. The number of leaves, the length and width of leaves, and the weight of pakchoi were significantly increased by applying fertilizer. The plant height in CM was significantly lower than that in CK. The number of leaves, leaf length and width, and weight were the highest in M.

The physicochemical parameters of pakchoi are shown in Figure 2C. The application of fertilizer significantly increased the TN, TP, and TK contents in the endosphere of pakchoi. The TOC content in the L treatment was significantly lower than that in CK. There was no significant difference in the contents of TOC, TN, and TK in the BSFL treatment (L, M, and H). The TP content in L was significantly higher than that in the other BSFL groups (M and H), but there was no significant difference compared with CM.

### TRG abundance In BSFL frass, rhizosphere soil, and pakchoi samples

**Figure 3:**
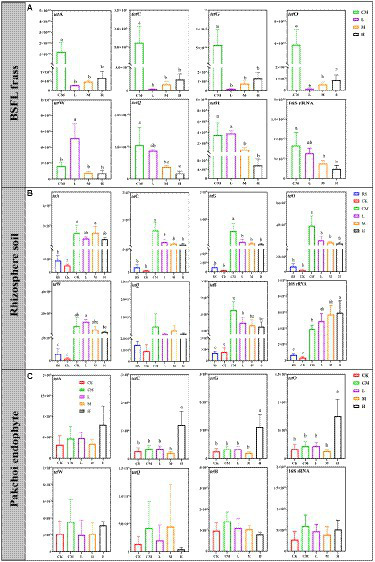
The absolute abundance of TRGs in BSFL frass **(A)**, rhizosphere soil **(B)**, and pakchoi endophytes **(C)** among different treatments. Significant differences between the means were determined by Tukey’s test. Differences were considered significant when *p* < 0.05, and different lowercase letters indicate significant differences. “RS” means initial pure soil, “CK” means untreated soil, “CM” means untreated manure without BSFL, “L” means 50 BSFL cultured in 100 g of fresh manure, “M” means 100 BSFL cultured in 100 g of fresh manure, “H” means 1,000 BSFL cultured in 100 g of fresh manure.

The reduction in TRGs by BSFL is related to the species of ARGs and rearing scale (Niu et al., 2022); as the rearing density of BSFL increases, it contributes to TRG reduction to a certain extent. We detected TRGs except for the *tet*T genes in all samples (Figure 3). The abundance of *tet*A, *tet*C, *tet*G and *tet*O in chicken manure was significantly reduced by BSFL treatment from 94.44 to 99.66% (Figure 3A), and the reduction of *tet*Q and *tet*H increased with the added density of BSFL additions. Interestingly, the abundance of *tet*W in L was significantly higher than that in the other groups.

A similar situation occurred in rhizosphere soil samples (Figure 3B). The abundances of *tet*C, *tet*G, *tet*O, and *tet*H in the rhizosphere soil treated with BSFL frass (L, M, and H) were significantly lower than those in CM. No significant differences were found in the abundance of *tet*A, *tet*W, and *tet*Q between the chicken manure and BSFL frass treatment groups. Notably, the abundance of TRGs except for *tet*A in M and H was not significantly different from that in RS and CK.

Regrettably, in the pakchoi endophyte samples from H, we detected a significantly higher abundance of *tet*C, *tet*G, and *tet*O than in other treatment groups (Figure 3C). This means that manure-borne ARGs could enter crops through the soil planting system during the application of BSFL frass, which undoubtedly has the potential to threaten human public health (Chen et al., 2017). Fortunately, the abundance of TRGs we detected in the pakchoi endophyte samples of the other treatment groups was not significantly different from that of the RS and CK groups (Figure 3C).

The total absolute abundance of TRGs is shown in Supplementary Figure S1. The application of BSFL frass by different rearing densities reduced the transmission of TRGs in manure to a large extent (92.40–96.66%; Supplementary Figure S1a), but the application risk of chicken manure (CM) and high-density BSFL frass (H) demonstrated that the abundance of TRGs in the rhizosphere soil after the application of chicken manure and H in the pakchoi endophyte samples was higher than that in the other groups (Supplementary Figures S1b,c). The total bacterial abundance in BSFL frass decreased linearly to a moderate extent with increasing BSFL application density (Supplementary Figure S1a).

The above results indicate that the application of high-density BSFL frass (H) to grow pakchoi may be a highly risky practice, led to the highest accumulation of TRGs in pakchoi samples.

### Microbial diversity

A total of 9,220 bacterial OTUs and 2,762 fungal OTUs were identified with 97% similarity. Overall, the bacterial and fungal diversity (Shannon) is shown in Supplementary Figures S2a,e, respectively. In general, the results of the Shannon index analysis showed that the microbial community (bacterial and fungal) richness and uniformity of endophytes in the pakchoi sample were significantly lower than those in the rhizosphere soil and BSFL frass samples. The bacterial diversity of rhizosphere soil samples was highest, and fungal diversity showed a decreasing trend with increasing BSFL density in BSFL frass or soil samples (Supplementary Figure S2e).

Principal coordinate analysis (PCoA) was used with the Bray–Curtis method to reveal the bacterial and fungal communication of BSFL frass, rhizosphere soil, and pakchoi endophyte samples (Supplementary Figures S2b–d, f–h). In the BSFL frass sample, regardless of the bacterial or fungal community, the community diversity among each treatment group was relatively independent (Supplementary Figures S2b,f). When fertilizer was applied, the microbial community diversity in the rhizosphere soil samples overlapped, but all were distinct from those in the unfertilized treatment group (Supplementary Figures S2c,g). In the pakchoi endophyte samples, the microbial community showed no significant difference (Supplementary Figures S2d,h).

### Microflora community composition

The compositions of the bacterial and fungal communities in BSFL frass, rhizosphere soil, and pakchoi endophyte samples were studied (Supplementary Figure S3). The bacterial community structures of the three types of samples were different. A total of 13 bacterial phyla are displayed in Supplementary Figure S3a. In the BSFL frass sample, the dominant phyla were Firmicutes and Bacteroides, accounting for 20.95–30.08% and 22.64–32.74% of the total bacteria, respectively, followed by Actinobacteriota, Spirochaetota, and Proteobacteria. In rhizosphere soil samples, the dominant phylum was Proteobacteria (25.31–46.12%), followed by unidentified Bacteria, Actinobacteriota, and Firmicutes. Almost all microorganisms in the endophyte of pakchoi after planting were Cyanobacteria (73.94–86.12%), followed by Proteobacteria (14.80–17.69%). The abundances of phyla Firmicutes and Bacteroides were significantly reduced in the process of producing pakchoi with BSFL frass, while Cyanobacteria abundance increased significantly (Supplementary Figure S2a).

A total of 21 bacterial genera are displayed in Supplementary Figure S3b. The BSFL frass samples contained many abundant genera of microorganisms, such as *Sphaerochaeta* (2.61–25.09%), *Rikenellaceae_RC9_gut_group* (0.07–18.55%), *Corynebacterium* (3.95–18.59%), *Acholeplasma* (1.25–7.10%), *Proteiniphilum* (2.03–5.13%), *Fermentimonas* (1.94–4.10%), *Halomonas* (0.20–10.02%), and *Oblitimonas* (0.15–10.67%). Compared with the BSFL frass samples, the relative abundance of microbial genera in the soil samples was lower, and the dominant genera was *Pseudomonas* (0.80–6.92%), followed by *Bacillus* (0.72–7.64%) and *Geitlerinema* (0.13–3.96%).

To gain insight into the microbial community structure of all samples, we also sequenced the fungal community, and the results showed that the fungal community structures of the three types of samples were more singular. A total of 11 fungal phyla are displayed in Supplementary Figure S3c. In the BSFL frass sample, the dominant fungal phylum was Ascomycota (50.91–91.18%). The dominant fungal phylum Ascomycota was significantly reduced to 43.14–63.37% in the rhizosphere soil samples. The relative abundance of the fungal phylum unidentified_Eukaryota, except in untreated soil, both before and after planting pakchoi (RS and CK) was 11.23–18.85%. In addition, the abundance of the fungal phylum Mucoromycota in the CM was significantly higher than that in any other treatment group by 29.97%.

A total of 15 fungal genera are displayed in Supplementary Figure S3d. The relative abundance of fungal genera in each treatment group fluctuated greatly. In the BSFL frass samples, the relative abundance of the fungal genus *Microascus* was significantly higher in H than in the others, reaching 64.34%. In addition, the dominant fungal genera were *Aspergillus* (24.92 and 42.77%) in L and H. In rhizosphere soil samples, the dominant fungal genus was *Phymatotrichopsis* (11.03–15.48%) in the BSFL application groups (L, M, and H). In addition, CM group had the highest proportion of a single fungal genus, *Mortierella,* among all the sample groups, reaching 29.10%.

### Correlation between TRGs, microflora, and physicochemical parameters

Redundancy analysis (RDA) was performed to further analyze the relationship between the TRGs and physicochemical parameters (TOC, TN, TP, TK, and THA) in BSFL frass and pakchoi samples (Supplementary Figures S4a,b). The results showed that 97.60% of the variance in physicochemical parameters could be explained by the selected variables with the first and second principal components in BSFL frass samples (Supplementary Figure S4a). This result showed that the analysis results could better reflect the impact of physicochemical parameters on TRGs. There was a strong positive correlation between the TN content and the abundance of *tet*A, *tet*C, *tet*G, and *tet*O, and the TP content was also strongly positively correlated with the abundance of *tet*Q and *tet*H, and the TOC content had a strong negative correlation with the abundance of *tet*W in BSFL frass samples. In addition, the first and second principal components explained 68.71 and 24.95% of the variation in physicochemical parameters by the selected variables in the pakchoi endophyte samples, respectively (Supplementary Figure S4b). There was a strong positive correlation between the TOC content and the abundance of *tet*Q, *tet*W, and *tet*H, and the TN and TP contents were also strongly negatively correlated with the abundance of all TRGs we detected the pakchoi endophyte samples (Supplementary Figure S4b).

A correlation diagram based on the relative abundances of TRGs and microflora (phylum level of bacterial and fungal) in BSFL frass, rhizosphere soil, and pakchoi endophyte samples is shown in Supplementary Figures S5a–c, respectively. The results showed that microflora was significantly correlated with TRGs. Interestingly, there was also a strong positive correlation between the abundance of *tet*A, *tet*C, *tet*G, and *tet*O among all samples.

The correlation between TRGs and microorganisms (phylum level) in BSFL frass samples is shown in Supplementary Figure S5a. The abundances of *tet*A, *tet*C, *tet*G and *tet*O were significantly positively correlated with the bacterial phylum Proteobacteria. The abundances of *tet*Q and *tet*H were significantly positively correlated with the bacterial phyla Firmicutes and Bacteroidota, respectively. The abundance of tetW was only significantly positively correlated with the bacterial phylum Bacteroidota, while the abundance of the bacterial phylum Spirochaetota was only significantly negatively correlated with *tet*H.

The correlation between TRGs and microflora (phylum level) in rhizosphere soil samples is shown in Supplementary Figure S5b. The abundance of the bacterial phylum Spirochaetota was significantly positively correlated with all the TRGs we detected except *tet*W. The abundance of *tet*W was significantly positively correlated with the bacterial phylum Bacteroidota. In addition, the abundance of fungal phyla was significantly negatively correlated with *tet*A, and the bacterial phylum Bacteroidota was significantly positively correlated with *tet*Q. The abundance of *tet*H was significantly negatively correlated with the bacterial phylum Actinobacteriota.

The correlation between TRGs and microflora (phylum level) in pakchoi endophyte samples is shown in Supplementary Figure S5c. Only the abundance of *tet*Q was significantly positively correlated with the abundance of the bacterial phylum Proteobacteria.

### Potential host microbes

**Figure 4:**
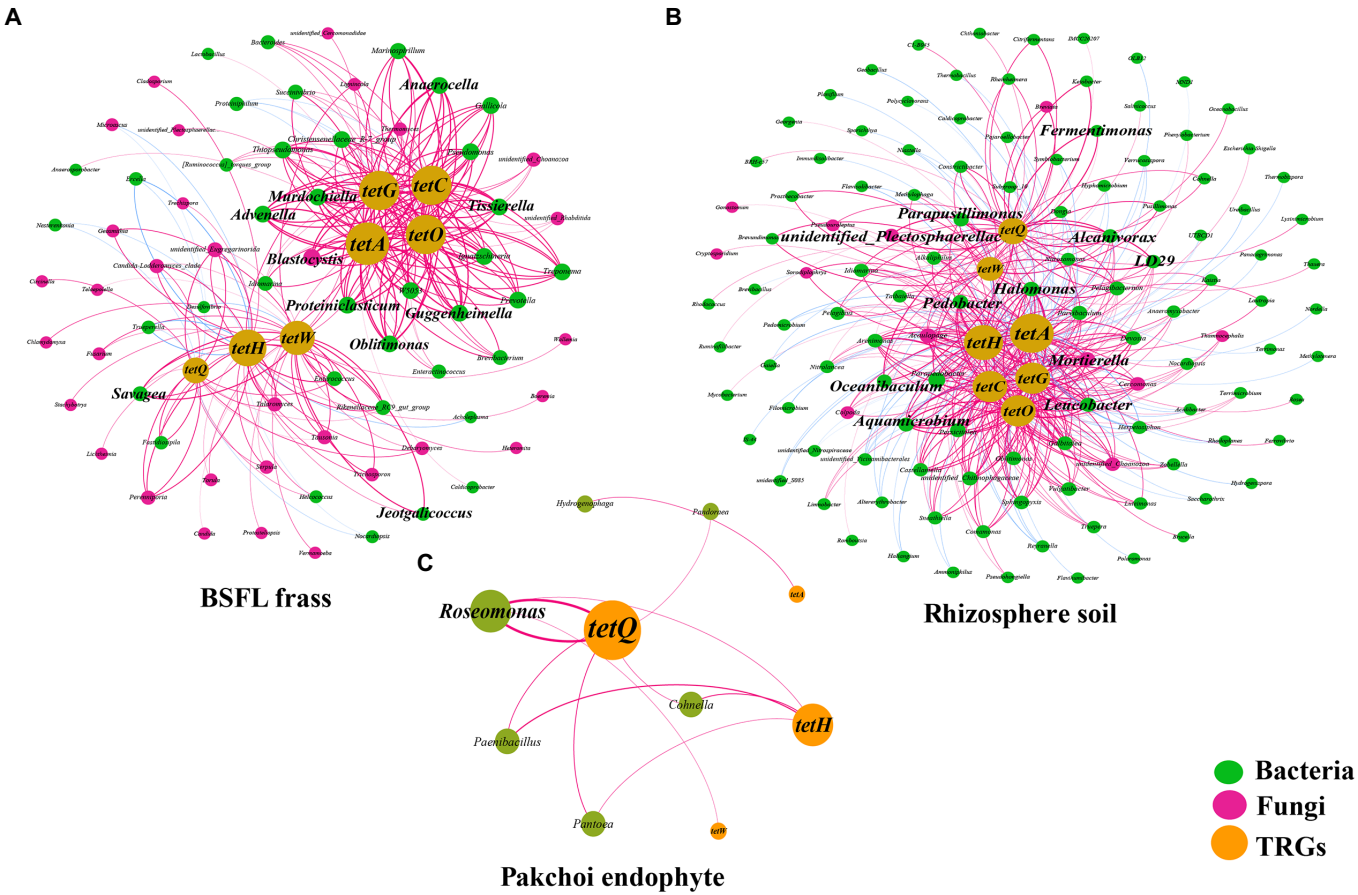
The network reveals the cooccurrence patterns between the detected TRGs and microbial taxa (genus level) in BSFL frass **(A)**, rhizosphere soil **(B)**, and pakchoi endophytes **(C)**. The nodes were colored according to the type of microorganism. The connection between TRGs and microorganism taxa in BSFL frass and rhizosphere soil samples represents a strong (Spearman’s correlation coefficient *p* > 0.8) and significant (*p* value <0.01) correlation. The connection between TRGs and microorganism taxa in the pakchoi endophyte sample represents a strong (Spearman’s correlation coefficient *p* > 0.6) and significant (*p* value <0.05) correlation. Node size is proportional to the number of connections; edge width is proportional to Spearman’s correlation coefficient value.

The co-occurrence patterns of TRGs and the microflora (bacterial and fungal) communities (genus level) in BSFL frass, rhizosphere soil, and pakchoi endophyte samples were studied using network analysis, as shown in Figure 4. In the BSFL frass samples, we found a total of 38 species of bacteria and 33 species of fungi as potential hosts for TRGs (Figure 4A). Interestingly, we found that *tet*A, *tet*C, *tet*G, and *tet*O had 22 common potential host bacteria and two common potential host fungi in the BSFL frass samples. We also found that *tet*W, *tet*Q, and *tet*H had three common potential host bacteria and seven common potential host fungi. Among these potential host microorganisms, we screened using the criterion of a correlation greater than 0.900, and the result showed that nine potential host bacteria (*Advenella*, *Anaerocella*, *Guggenheimella*, *Jeotgalicoccus*, *Murdochiella*, *Oblitimonas*, *Proteiniclasticum*, *Savagea*, and *Tissierella*) and one potential host fungus (*Blastocystis*) had a strong correlation with TRGs.

In the rhizosphere soil samples, we found more complex network relationships between microflora and TRGs, with a total of 109 species of bacteria and 11 species of fungi as potential host bacteria for TRGs (Figure 4B). We found that *tet*A, *tet*C, *tet*G, *tet*O, and *tet*H had 24 common potential host bacteria and four common potential host fungi in rhizosphere soil samples. Among these potential host microorganisms, we screened using the criterion of a correlation coefficient greater than 0.900, and the results showed that nine potential host bacteria (*Alcanivorax*, *Aquamicrobium*, *Fermentimonas*, *Halomonas*, *LD29*, *Leucobacter*, *Oceanibaculum*, *Parapusillimonas*, and *Pedobacter*) and two potential host fungi (*Mortierella* and *unidentified_Plectosphaerellaceae*) had a strong correlation with TRGs.

In pakchoi endophyte samples, we found few network relationships between microflora and TRGs, with only six species of bacteria as potential host bacteria for TRGs (Figure 4C). We found that *tet*H and *tet*Q had four common potential host bacteria in pakchoi endophyte samples. Due to the lack of network correlation, we lowered the screening criteria to a correlation of 0.800 and found only that *Roseomonas* had a strong correlation with TRGs.

**Figure 5:**
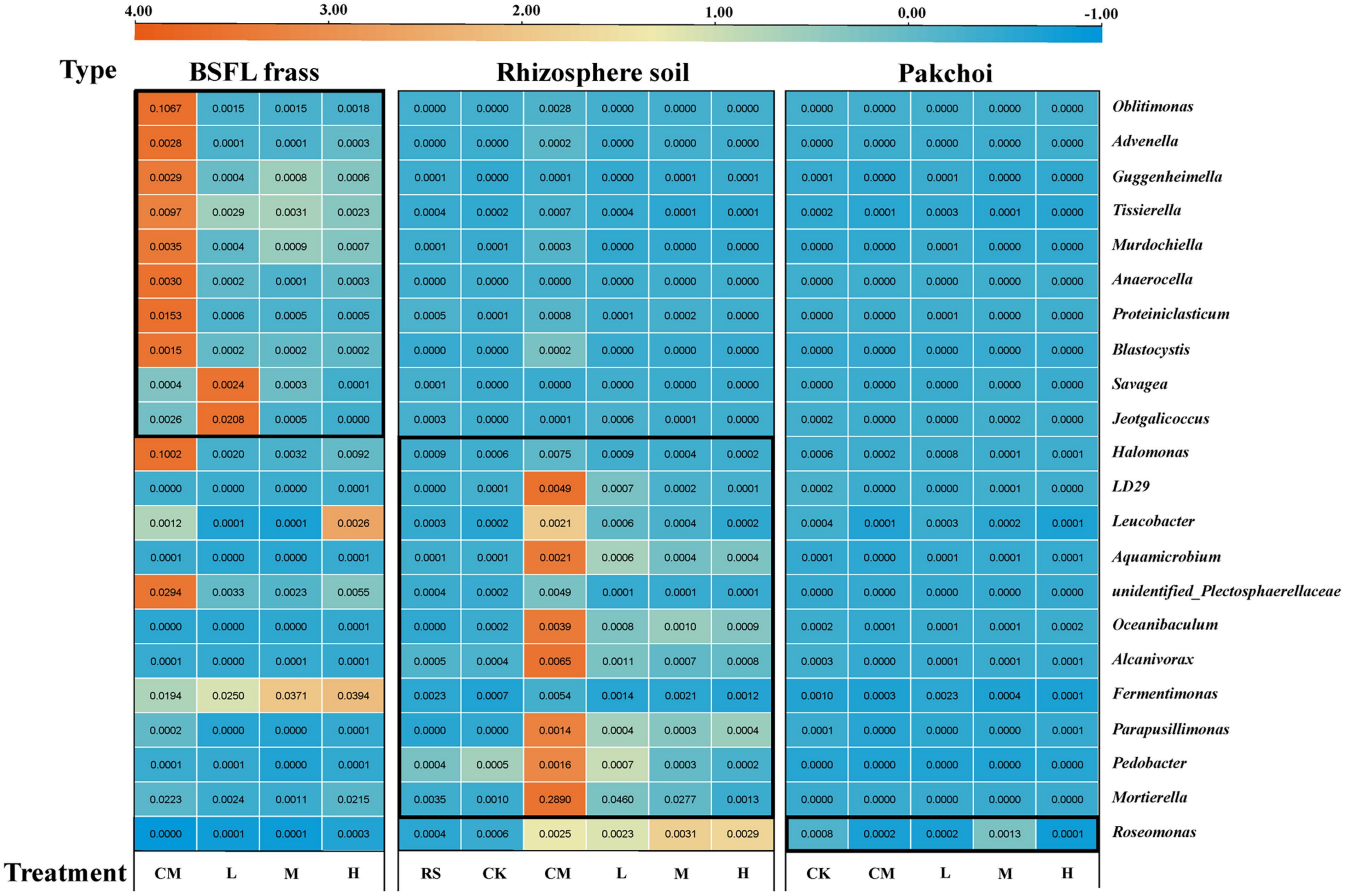
Relative abundances of 22 potential host microorganisms in BSFL frass, rhizosphere soil, and pakchoi endophytes with high correlation (correlation at least greater than 0.9, except for pakchoi endophyte samples, which are only greater than 0.8) among different treatments. “RS” means initial pure soil, “CK” means untreated soil, “CM” means untreated manure without BSFL, “L” means 50 BSFL cultured in 100 g of fresh manure, “M” means 100 BSFL cultured in 100 g of fresh manure, “H” means 1,000 BSFL cultured in 100 g of fresh manure.

Subsequently, we analyzed the relative abundance of the screened microorganisms (20 bacteria and 2 fungi) that were highly correlated with TRGs (Figure 5). Even if the potential host microorganisms of TRGs of different sample types did not appear to merge, we observed that the host microorganisms were mainly concentrated in the BSFL frass and rhizosphere soil samples in the CM. Therefore, we paid more attention to the potential TRG host microorganisms that appeared in L, M, and H. Finally, we focused on *Oblitimonas* (the highest total abundance in all treatments) and *Tissierella* (the highest total abundance in the BSFL treatment) in the BSFL frass samples. The same screening method was used to select *Mortierella* and *Fermentimonas* in the rhizosphere soil samples and *Roseomonas* in the pakchoi samples.

**Figure 6:**
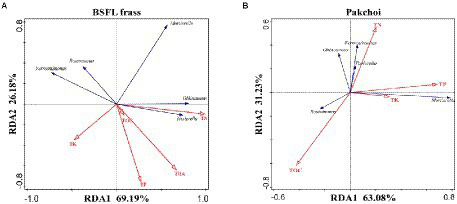
Redundancy analysis (RDA) of the potential host microorganism (*Oblitimonas*, *Tissierella, Mortierella*, *Fermentimonas*, and *Roseomonas*) patterns of BSFL frass **(A)** and pakchoi **(B)** using chemical properties as explanatory variables.

We performed RDA on the OTUs of these five microorganisms and the physicochemical factors of BSFL frass and pakchoi samples (Figure 6). The results showed that 95.37% of the variance in physicochemical parameters could be explained by the selected variables with the first and second principal components in BSFL frass samples (Figure 6A). This result showed that the analysis results could better reflect the impact of physicochemical parameters on potential host microorganisms. A strong positive correlation was found between the TN content and the absolute abundance of *Oblitimonas* and *Tissierella*. The TOC, TP, and THA contents had a strong negative correlation with *Fermentimonas* and *Roseomonas*. The only fungal microorganism (*Mortierella*) was significantly negatively correlated with the TK content. In addition, the first and second principal components explained 63.08 and 31.23% of the variation in physicochemical parameters by the selected variables in the pakchoi endophyte samples, respectively (Figure 6B). A strong positive correlation was found between *Roseomonas* and TOC; similarly, *Mortierella* was also strongly positively correlated with the TP and TK contents. Interestingly, a strong positive correlation was found between the TN content and the absolute abundance of *Oblitimonas*, *Tissierella,* and *Fermentimonas*.

### Application value and risk assessment of BSFL frass

**Figure 7:**
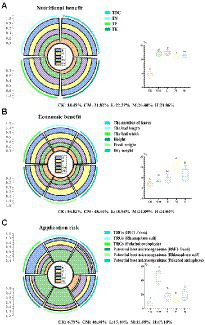
The best larval density group analysis. Nutritional benefits **(A)**, economic benefits **(B)**, and application risks **(C)** were considered. Each part of the pie represents one influencing factor, the different color blocks in each influencing factor indicate each larval density, and the proportion of each block indicates the ratio of each larval density to the influencing factor. Significant difference analysis is depicted to the right of each consideration, and different letters indicate significant differences. Significant differences between the means were determined by Tukey’s test. Differences were considered significant when *p* < 0.05, and different lowercase letters indicate significant differences. “RS” means initial pure soil, “CK” means untreated soil, “CM” means untreated manure without BSFL, “L” means 50 BSFL cultured in 100 g of fresh manure, “M” means 100 BSFL cultured in 100 g of fresh manure, “H” means 1,000 BSFL cultured in 100 g of fresh manure.

To select the BSFL addition density with the best application effect and the lowest application risk, a computational model was used to evaluate the appropriate density of larval addition in this paper (Figure 7). We mainly considered the pakchoi growth index as an indicator of economic benefit (Figure 3B); the TOC, TN, TP, and TK contents of pakchoi as nutritional benefit factors (Figure 3C); and the abundance of TRGs and highly correlated potential host microbes as indicators of application risk (Figures 2, 5). Finally, we considered all factors to select the optimal larval density. The application of chicken manure or BSFL frass both contributed significantly to the improvement of the nutritional value of pakchoi (21.06–22.27%; Figure 7A), and the highest contribution was in the L group, with a value only 1.87% higher than that in the M group and the primer sequences are shown in (*p <* 0.05). In terms of the economic benefit contribution, the M group had an absolute advantage of 24.89% (*p* < 0.05) over the other treatment groups. In terms of application risk, the application of chicken manure had the highest risk, reaching 46.98% (*p* < 0.05), while the rest of the treatment groups had no significant difference compared with the CK group, and the M group had the lowest risk of 11.95% (*p >* 0.05).

## Discussion

### Nutritional and economic value of pakchoi and residual TRGs

BSFL frass can provide sufficient nutrition for the growth of pakchoi similar to chicken manure (Figures 3A,C), but the nutrient content between them is not completely consistent. Similarly, BSFL frass applied to corn (Garttling et al., 2020), sweet potato (Romano et al., 2022), vegetables, and herbs (Borkent and Hodge, 2021) has the potential to be an effective organic fertilizer. Although the total nitrogen content in BSFL frass was lower than that in chicken manure (Figure 3A), this may be because nitrogen is a key factor in the transformation of BSFL crude protein during growth. However, an increase in the amount of fertilizer may cause negative effects. Borkent and Hodge (2021) used BSFL frass as a fertilizer to grow basil, lettuce, and parsley and found that excessive application of this fertilizer resulted in restricted root development.

To further understand the risk of BSFL frass application, we detected 8 ARGs and 16S rRNA genes in all samples, among which the *tet*T gene was never detected. This result is not completely consistent with those of a previous study (Cai et al., 2018a), although this scholar used tetracyclines to induce TRGs. In this study, BSFL could indeed reduce 92.40–96.66% of TRGs detected in chicken manure. Similarly, other scholars have found that it can reduce *mcr*-1 by 89.16–99.52% in chicken manure (Niu et al., 2022). The remaining TRGs after BSFL treatment did not have a significant impact on rhizosphere soil, but the direct application of chicken manure could significantly increase the absolute abundance of TRGs in rhizosphere soil (Supplementary Figure S1; Figure 7C). In this view, the application of BSFL frass was safer and more reliable than the application of manure. In particular, the application of chicken manure did not affect the TRGs in pakchoi endophytes. Studies have shown that the application of pig manure increased the accumulation of ARGs in the skin of carrots (Mei et al., 2021). Sun et al. (2021) showed that the application of beef cattle manure to surface soil planted with lettuce can cause antibiotic-resistant bacteria to enter the phyllosphere from the surface soil, thereby causing the transmission of ARGs. Interestingly, we found that the absolute abundance of TRGs, especially *tet*A, *tet*C, and *tet*G, in the pakchoi endophytes was significantly increased after the application of BSFL frass from a higher proportion of larvae added (H). Obviously, the BSFL rearing scale could affect the risk of BSFL frass application, and this result was similar to those of Niu et al. (2022) For the accumulation of TRGs alone, in this paper, BSFL frass produced at a lower rearing scale (L and M) was a sustainable product that was safer than the direct application of chicken manure.

### Potential host microorganisms for TRGs

We explored the application risk of BSFL frass from the perspective of the microbiome. During the production of BSFL frass, the absolute abundance of total bacteria in the product was decreased as the rearing scale increases (Figure 3A). Although we excluded the option of BSFL frass produced on a high-density rearing scale (H), after application of BSFL frass produced in this mode, the total bacterial abundance in rhizosphere soil and the abundance of TRGs in pakchoi endophytes once again confirmed that our judgment was correct.

From the correlation analysis of microorganisms and TRGs, Proteobacteria, Spirochaetota, Bacteroidota, and Firmicutes were potential host bacteria (phylum level) of TRGs in BSFL frass and rhizosphere soil. However, reports have been published of Proteobacteria as ARG hosts are common (Wang et al., 2020; Qian et al., 2021; Yanlong et al., 2021; Zhang et al., 2021). We performed network analysis on the relative abundance of microorganisms at the genus level and their corresponding TRGs from different samples and found that rhizosphere soil had the most complex association (Figure 4) and was associated with the highest microbial species (Supplementary Figures S2a,e).

In the BSFL frass samples, the host bacteria of the TRGs we detected were mostly Firmicutes (*Guggenheimella*, *Murdochiella*, *Proteiniclasticum*, *Tissierella*, *Jeotgalicoccus*, and *Savagea*), and the host fungi were Eukaryota (*Blastocystis*). However, combined with the previous correlation analysis, we focused more on the microorganisms in Proteobacteria, especially *Advenella* and *Oblitimonas*. Notably, *Advenella* and *Oblitimonas* are clinically pathogenic microorganisms (Church et al., 2020). Fortunately, these two microbes were almost undetectable in pakchoi endophyte samples and were significantly reduced in BSFL frass after digestion with BSFL. Finally, combined with the results of the relative abundance heatmap analysis (Figure 5), we identified *Tissierella* and *Oblitimonas* as the host bacteria of *tet*A, *tet*O, *tet*C, and *tet*G in the BSFL frass samples. The genus *Tissierella* was first named by Colins and Shah (1986). A recent study showed that *Tissierella* is a potential host of ARGs in lignite-treated pig manure (Guo et al., 2020a). The results of that study were similar to those of the present study despite differences in manure type and treatment. Few reports exist about the genus *Oblitimonas*. Drobish et al. (2016) first isolated the strains from eight patients and named them *Oblitimonas*. A study reported that the tet(31) tetracycline resistance determinant was commonly found in *Oblitimonas alkaliphila* isolated from farm animals and related environments (Shi et al., 2019). Even recent reports suggest merging *Oblitimonas* with *Thiopseudomonas* (Rudra and Gupta, 2021). Although we first focused on Spirochaetota in rhizosphere soil samples, we shifted our attention to Bacteroidota due to the lack of genus-level microorganisms in Spirochaetota. Combining the network analysis and heatmap analysis results, we identified *Mortierella* and *Fermentimonas* as the host bacteria of *tet*H and *tet*Q, respectively, in the rhizosphere soil sample. *Mortierella* are plant growth-promoting fungi and are found in rhizosphere soil (Li et al., 2018; Ozimek and Hanaka, 2021). These fungi have great potential in the degradation of pesticides (Ellegaard et al., 2013) and heavy metal bioremediation (Cui et al., 2017). there was less research that fungi transmit ARGs currently exists. *Fermentimonas*, like *Oblitimonas*, is a new genus recently discovered (Maus et al., 2017), and few reports have been published about its association with ARGs. Similarly, although the network analysis plots showed that pakchoi endophytes were less associated with TRGs, we still used the same method to target *Roseomonas* as the host bacteria of *tet*Q in the pakchoi endophyte sample, although the abundance of this bacteria was not high. *Roseomonas* was detected in a constructed wetland as an extended-spectrum beta-lactamase bacterium associated with a clinical infection and is a potential host of intl1 (Henderson et al., 2022). *Roseomonas* is reportedly a clinical pathogenic microorganism (Church et al., 2020), and even if the relative abundance of this microorganism is less than 0.1%, it is a threat to public health.

### Key abiotic factors affecting TRG abundance and potential host microorganisms

We conducted an association analysis between abiotic factors and TRGs and potential host microorganisms to identify factors that were closely related. From the results of the redundancy analysis (Supplementary Figure S4; Figure 6), the key factors of ARGs and potential host microorganisms in different samples were not necessarily consistent. For example, in the BSFL frass sample, the TN, TOC, and THA contents were found to have the greatest positive effect on *tet*A, *tet*O, *tet*C, and *tet*G, while the TP content had the greatest positive effect on *tet*Q and *tet*H (Supplementary Figure S4a). Similarly, we found a significant positive correlation with *Oblitimonas* and *Tissierella* at the same time (Figure 6A). However, the remaining three potential host microorganisms did not appear to correspond. Interestingly, both TN and TP contents had an inverse relationship with all TRGs tested in the pakchoi endophyte sample, while the TOC content still had a positive effect on TRGs, albeit only for *tet*Q, *tet*W, and *tet*H (Supplementary Figure S4b). The difference was that the TK and TP contents were positively correlated with *Mortierella*; the TN contents were positively correlated with *Oblitimonas*, *Fermentimonas*, and *Tissierella*; and the TOC contents were positively correlated with *Roseomonas* (Figure 6B). Similar to the occurrence of BSFL frass samples, the TOC content in pakchoi samples was only consistent with the occurrence of *Roseomonas* and corresponding TRGs. These two phenomena demonstrate that the host microorganisms of TRGs were changed in different sample types. At the same time, *Oblitimonas* and *Tissierella* were related to TN content, and *Roseomonas* was related to TOC content. This result is consistent with previous studies showing that TOC may participate in regulating the occurrence and distribution of ARGs (Guo et al., 2020b). Shen et al. (2022) reported that the TN content was the key factor controlling ARG pollution in groundwater. Similarly, Fan et al. (2021) found that the contents of TN and TP in paddy fields were closely related to the distribution of dominant ARGs. BSFL reduced the abundance of TRG host microorganisms (mainly *Oblitimonas* and *Tissierella*) by converting nitrogen in chicken manure into crude protein, thereby reducing the abundance of *tet*A, *tet*O, *tet*C, and *tet*G carried by them. These microbes eventually almost disappeared and did not appear in the pakchoi endophyte (Figure 5). Likewise, although the *Roseomonas* correlation was high, the relative abundance remained at a very low level.

### Optimal BSFL addition rate

With the increase in the proportion of BSFL added, the M group (100 BSFL cultured in 100 g of fresh manure) had the optimal addition rate among the treatment groups with different BSFL addition rates. This group had the significantly highest contribution to economic benefits (24.89%), with only a 1.87% reduction in nutritional value contribution compared with the L group, and it had the highest nutritional value contribution rate and the lowest contribution (11.95%) to the applied risk value; thus, this was the optimal larval addition rate.

## Conclusion

In summary, the treatment of chicken manure by BSFL is a sustainable and economically promising practice in the current manure treatment process. How to utilize the treated BSFL frass requires more research from multiple aspects. In this study, only pakchoi was grown on BSFL frass produced with different rearing scales, and the risk of TRG transmission and the distribution of potential host microorganisms were evaluated. The results confirmed that BSFL frass produced at the rearing scale of 100 BSFL cultured in 100 g of fresh manure can improve the growth performance of pakchoi and has a low risk of TRG transmission to pakchoi.

## Data availability statement

The datasets presented in this study can be found in online repositories. The names of the repository/repositories and accession number(s) can be found at: https://www.ncbi.nlm.nih.gov/, SRP390589.

## Author contributions

JC: data analysis, figure plotted, and manuscript writing. YC: conducted experiments, data analysis, and figure plotted. WD: conducted experiments and data analysis. SX: Experiment design, manuscript editing, project administration, and funding acquisition. XL: Project administration and funding acquisition. All authors contributed to the article and approved the submitted version.

## Funding

This study was supported by the National Natural Science Foundation of China (32072783), the Science and Technology Program of Guangdong province, China (2020B1212060060) and the earmarked fund for the Modern Agro-industry Technology Research System (CARS-40).

## Conflict of interest

The authors declare that the research was conducted in the absence of any commercial or financial relationships that could be construed as a potential conflict of interest.

## Publisher’s note

All claims expressed in this article are solely those of the authors and do not necessarily represent those of their affiliated organizations, or those of the publisher, the editors and the reviewers. Any product that may be evaluated in this article, or claim that may be made by its manufacturer, is not guaranteed or endorsed by the publisher.

## Footnotes

^1^ http://ccb.jhu.edu/software/FLASH/

^2^ http://qiime.org/scripts/split_libraries_fastq.html

^3^ http://www.drive5.com/usearch/manual/uchime_algo.html
