# Supplementary material for: Transmission of tetracycline resistance genes and microbiomes from manure-borne black soldier fly larvae frass to rhizosphere soil and pakchoi endophytes
Source: Front Microbiol. 2022 Oct 31;13:1014910. doi: 10.3389/fmicb.2022.1014910 (PMC9659867; doi:10.3389/fmicb.2022.1014910)
Supplement: Supplementary file 1 [file Data_Sheet_1.docx]

**Supplementary Information**

**Transmission of tetracycline resistance genes and microbiomes from manure-borne black soldier fly larvae frass to rhizosphere soil and pakchoi endophytes**

Jingyuan Chen^a^, Yingfeng Cai^a^, Weikang Deng^a^, Sicheng Xing^a,b,c^ and Xindi Liao^a,b,c^*

^a^ College of Animal Science, South China Agricultural University, Guangzhou 510642, Guangdong, China

^b^ Guangdong Provincial Key Lab of Agro-Animal Genomics and Molecular Breeding, and Key Laboratory of Chicken Genetics, Breeding and Reproduction, Ministry Agriculture, Guangzhou 510642, Guangdong, China

^c^ National-Local Joint Engineering Research Center for Livestock Breeding, Guangzhou 510642, Guangdong, China

*** Corresponding author**

To whom correspondence should be addressed Tel: +86 20 85288229, Fax: +86 20 85280203. E-mail: [xdliao@scau.edu.cn](mailto:xdliao@scau.edu.cn).

**Table S1** The primer sequences of TRGs tested in this paper.

| Gene name | Primer (5′-3′) | | Size | References |
| --- | --- | --- | --- | --- |
| *tet*A | Forward | CAGGCAGGTGGATGAGGAA | 174 | (Huang et al., 2015) |
|  | Reverse | GGCAGGCAGAGCAAGTAGAG |  |  |
| *tet*C | Forward | TGCAACTCGTAGGACAGGTG | 140 | (Huang et al., 2015) |
|  | Reverse | ACCAGTGACGAAGGCTTGAG |  |  |
| *tet*G | Forward | GCAGAGCAGGTCGCTGG | 134 | (Aminov et al., 2001) |
|  | Reverse | CCYGCAAGAGAAGCCAGAAG |  |  |
| *tet*O | Forward | ACGGARAGTTTATTGTATACC | 171 | (Aminov et al., 2001) |
|  | Reverse | TGGCGTATCTATAATGTTGAC |  |  |
| *tet*Q | Forward | AGAATCTGCTGTTTGCCAGTG | 169 | (Aminov et al., 2001) |
|  | Reverse | CGGAGTGTCAATGATATTGCA |  |  |
| *tet*W | Forward | GAGAGCCTGCTATATGCCAGC | 168 | (Aminov et al., 2001) |
|  | Reverse | GGGCGTATCCACAATGTTAAC |  |  |
| *tet*H | Forward | CAACCCATTACGGTGTGCTA | 164 | (Szczepanowski et al., 2009) |
|  | Reverse | AAGTGTGGTTGAGAATGCCA |  |  |
| *tet*T | Forward | CCATATAGAGGTTCCACCAAATCC | 73 | (Hu et al., 2018) |
|  | Reverse | TGACCCTATTGGTAGTGGTTCTATTG |  |  |
| 16S rRNA | Forward | GTGSTGCAYGGYTGTCGTCA | 146 | (Rafraf et al., 2016) |
|  | Reverse | ACGTCRTCCMCACCTTCCTC |  |  |

**
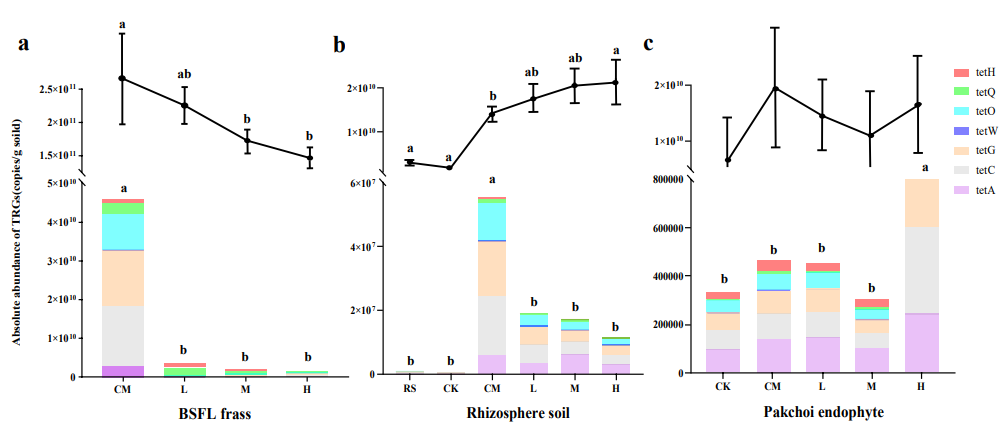
**

**Figure S1.** The absolute abundance stacking map of TRGs in BSFL frass (a), rhizosphere soil (b), and pakchoi endophytes (c) among different treatments. The column type was expressed as the absolute abundance stacking map of total TRGs (detected in this paper), and the line was expressed as the absolute abundance of total bacteria. Significant differences between the means were determined by Tukey’s test. Differences were considered significant when *P* < 0.05, and different lowercase letters indicate significant differences. “RS” means initial pure soil, “CK” means untreated soil, “CM” means untreated manure without BSFL, “L” means 50 BSFL cultured in 100 g of fresh manure, “M” means 100 BSFL cultured in 100 g of fresh manure, “H” means 1000 BSFL cultured in 100 g of fresh manure.


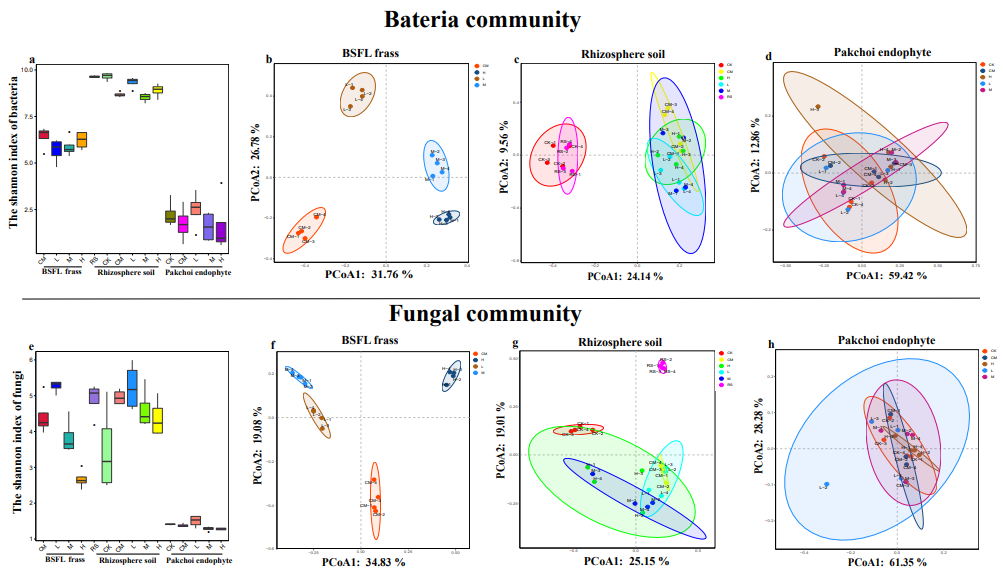


**Figure S2.** Alpha and beta analyses of bacteria and fungi. a) Shannon index of bacteria; b-d) PCoA1 analysis of bacteria in BSFL frass, rhizosphere soil, and pakchoi endophytes; e) Shannon index of fungi; f-h) PCoA1 analysis of fungi in BSFL frass, rhizosphere soil, and pakchoi endophytes. “RS” means initial pure soil, “CK” means untreated soil, “CM” means untreated manure without BSFL, “L” means 50 BSFL cultured in 100 g of fresh manure, “M” means 100 BSFL cultured in 100 g of fresh manure, “H” means 1000 BSFL cultured in 100 g of fresh manure.


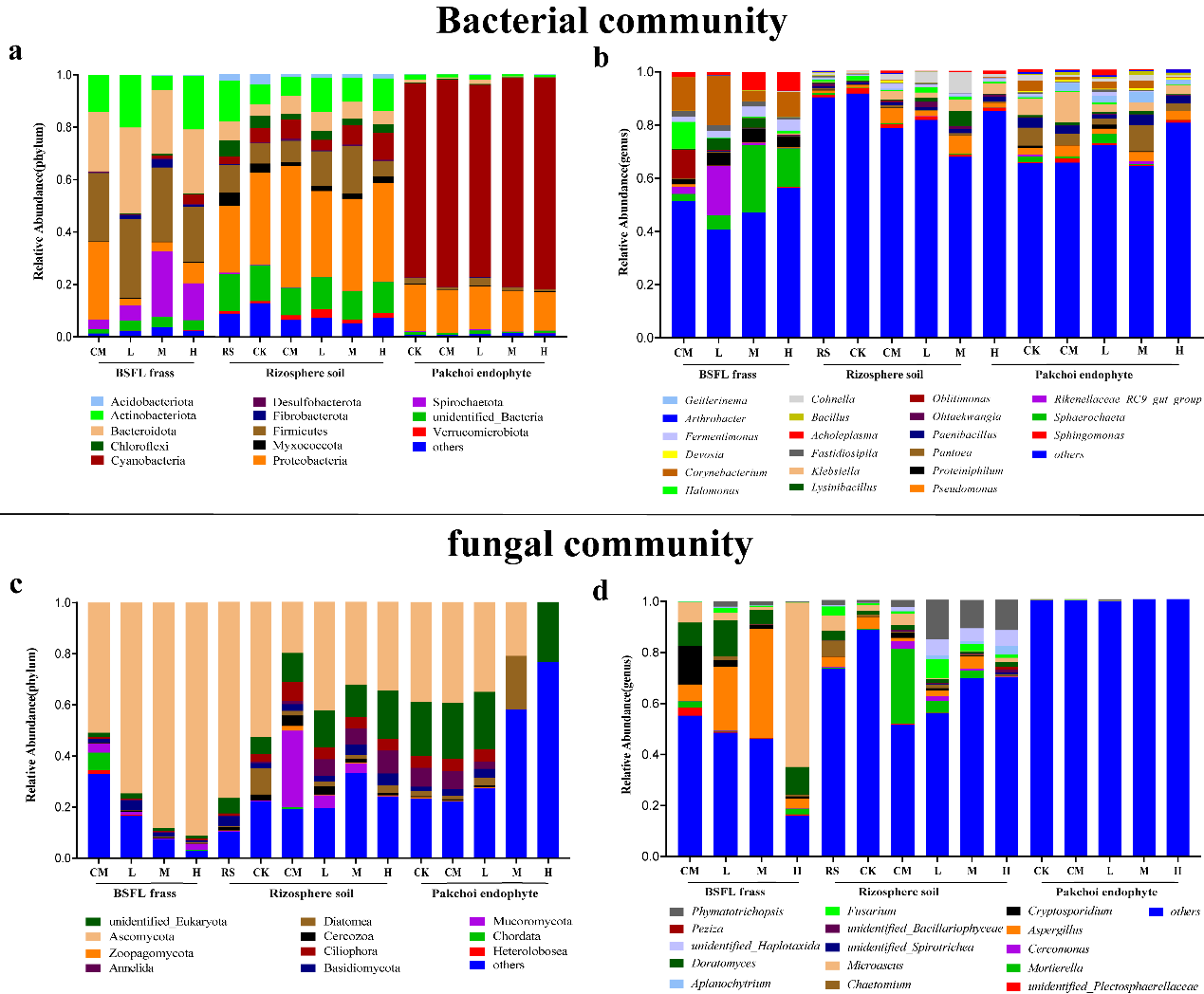


**Figure S3.** Relative abundance of bacterial and fungal communities at the phylum (a, c) and genus levels (b, d) in BSFL frass, rhizosphere soil, and endophytes of pakchoi among different treatments. “RS” means initial pure soil, “CK” means untreated soil, “CM” means untreated manure without BSFL, “L” means 50 BSFL cultured in 100 g of fresh manure, “M” means 100 BSFL cultured in 100 g of fresh manure, “H” means 1000 BSFL cultured in 100 g of fresh manure.


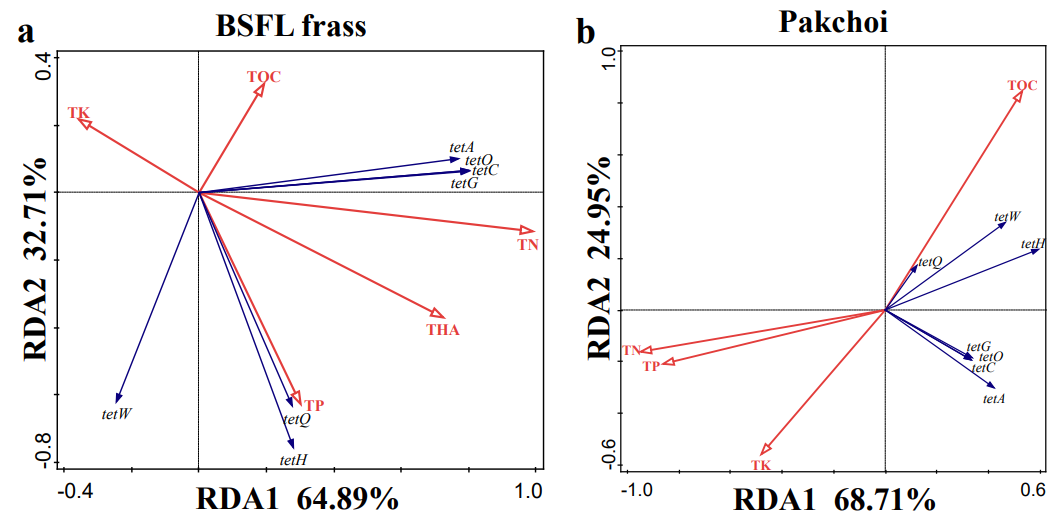


**Figure S4.** Redundancy analysis (RDA) of the TRG patterns of BSFL frass (a) and pakchoi (b) using chemical properties as explanatory variables. TOC (total organic carbon), TN (total nitrogen), TP (total phosphorus), TK (total potassium), and THA (total humic acid).


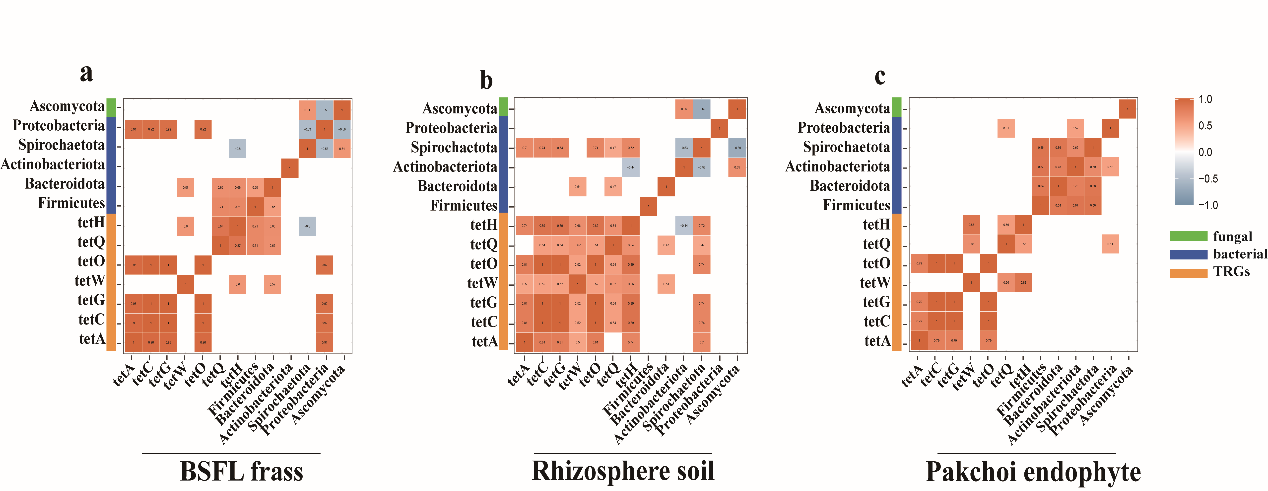


**Figure S5.** Correlation analysis of bacterial and fungal communities with TRGs of BSFL frass (a), rhizosphere soil (b), and endophytes of pakchoi (c).

**References:**

Aminov, R.I., Garrigues-Jeanjean, N., Mackie, R.I., 2001. Molecular Ecology of Tetracycline Resistance: Development and Validation of Primers for Detection of Tetracycline Resistance Genes Encoding Ribosomal Protection Proteins. APPLIED AND ENVIRONMENTAL MICROBIOLOGY 67, 22-32.

Hu, H.W., Wang, J.T., Singh, B.K., Liu, Y.R., Chen, Y.L., Zhang, Y.J., He, J.Z., 2018. Diversity of herbaceous plants and bacterial communities regulates soil resistome across forest biomes. ENVIRONMENTAL MICROBIOLOGY 20, 3186-3200.

Huang, M., Zhang, W., Liu, C., Hu, H., 2015. Fate of trace tetracycline with resistant bacteria and resistance genes in an improved AAO wastewater treatment plant. PROCESS SAFETY AND ENVIRONMENTAL PROTECTION 93, 68-74.

Rafraf, I.D., Lekunberri, I., Sanchez-Melsio, A., Aouni, M., Borrego, C.M., Balcazar, J.L., 2016. Abundance of antibiotic resistance genes in five municipal wastewater treatment plants in the Monastir Governorate, Tunisia. ENVIRONMENTAL POLLUTION 219, 353-358.

Szczepanowski, R., Linke, B., Krahn, I., Gartemann, K., Gützkow, T., Eichler, W., Pühler, A., Schlüter, A., 2009. Detection of 140 clinically relevant antibiotic-resistance genes in the plasmid metagenome of wastewater treatment plant bacteria showing reduced susceptibility to selected antibiotics. MICROBIOLOGY 155, 2306-2319.
